# Kinome Profiling

**DOI:** 10.6064/2012/306798

**Published:** 2012-08-07

**Authors:** Maikel P. Peppelenbosch

**Affiliations:** Department of Gastroenterology and Hepatology, Erasmus MC University Medical Center Rotterdam, L-459, P.O. Box 2040, NL-3000 CA Rotterdam, The Netherlands

## Abstract

The use of arrays in genomics has led to a fast and reliable way to screen the transcriptome of an organism. It can be automated and analysis tools have become available and hence the technique has become widely used within the past few years. Signal-transduction routes rely mainly on the phosphorylation status of already available proteins; therefore kinases are central players in signal-transduction routes. The array technology can now also be used for the analysis of the kinome. To enable array analysis, consensus peptides for kinases are spot on a solid support. After incubation with cell lysates and in the presence of radioactive ATP, radioactive peptides can be visualized and the kinases that are active in the cells can be determined. The present paper reviews comprehensively the different kinome array platforms available and results obtained hitherto using such platforms. It will appear that this technology does not disappoint its high expectations and is especially powerful because of its species independence. Nevertheless, improvements are still possible and I shall also sketch future possible directions.

## 1. Introduction

Over the last 15 years array and mass spectrometry technologies have enabled the determination of the transcriptome [1–3] and proteome [4–6] of biological samples. This information is of significant value to our elucidation of the molecular mechanisms that govern cellular physiology. However, an equally, if not more, important goal is to define those proteins that participate in signaling pathways that are active in cells [7, 8]. Enzymes that phosphorylate tyrosine, serine and threonine residues on other proteins (kinases [9]) play a major role in signaling cascades that determine cell cycle entry, survival, and differentiation fate in the tissues of the body [10]. Most members of the kinase superfamily of enzymes can be recognized from the primary sequence by the presence catalytic eukaryotic protein kinase (ePK) domain of approximately 250 amino acids, whereas a much smaller number of protein kinases do not share this catalytic domain and are often collectively called atypical kinases [11]. A comparison of the kinase domains both within and between species displays substantial diversity, which is further complicated by the noncatalytic functional domains of kinases involved in regulation, interactions with other protein partners or the subcellular localisation of kinases. Together, the diversity in catalytic and noncatalytic domains explains to high degree the functional diversification of kinases within the eukaryotic kingdom. Eukaryotic kinases are now generally classified in seven major groups: the cyclic nucleotide- and Ca^2+^-/phospholipid-dependent kinases; a group consisting of the cyclin-dependent and cyclin-dependent-like kinases, mitogen-activated kinases, and glycogen synthase kinases; the tyrosine kinases; the tyrosine kinase-like group (which are in fact serine/threonine protein kinases; the calmodulin-dependent kinases; the casein kinase 1 group and the STE group (STE is a contraction of sterile, reflecting the fact that genes belonging to this group were first identified in the analysis of sterile yeast mutants) and which includes the enzymes acting upstream of the mitogen-activated kinases [12, 13]. In particular, knowing which kinases signaling pathways are being utilized in specific cells is of critical importance for understanding biology and pathology and for treating disease [14, 15]. 

Traditional genetic and biochemical approaches can certainly provide answers here, but for technical and practical reasons there is typically pursued one gene or pathway at a time. Thus, a more comprehensive approach is needed in cells during health and disease. Towards this end, kinome analysis techniques have been developed and their application has started to provide worthwhile insights in the biology of plethora of experimental and clinical systems [16–18].

## 2. Development of Kinome Technologies

Massive parallel analysis using array technology has become the mainstay for the analysis of genomes and transcriptomes. Since the determination of the transcriptome, the understanding of cellular functioning has improved dramatically. Novel insights have led to the notion that the majority of the transcriptome is necessary to keep a cell functioning and could be regarded as the minimal transcriptome. Only a small portion of the transcripts present in the cell determines the identity of the cell and these critical transcripts are expressed at low levels [19, 20]. Therefore small changes in the expression profiles in the transcriptome can lead to large changes in enzymatic profile of the cell leading to significant differences in cell functioning. Thus, a comprehensive description of cellular metabolism may be more useful than such a description of the genome and transcriptome [17, 21]. 

Early this century, important progress has been made to adapt array technology to measure enzymatic activity in whole cell lysates with the preparation of protein chips for the assessment of protein substrate interactions and the generation of peptide chips for the appraisal of ligand-receptor interactions and enzymatic activities [22]. Houseman et al. showed that employing peptide chips, prepared by the Diels-Alder-mediated immobilization of one kinase substrate (for the nonreceptor tyrosine kinase c-Src) on a monolayer of alkanethiolates on gold, allows quantitative evaluation of kinase activity [23]. Hence, in principle an array exhibiting specific consensus sequences for protein kinases across the entire kinome (the combined activity of all cellular kinases) allows a comprehensive detection of signal transduction events in whole cell lysates and indeed we were able to demonstrate the usefulness of such peptide arrays for studying signal transduction as was demonstrated by the generation of the first comprehensive description of the temporal kinetics of phosphorylation events induced by lipopolysaccharide stimulation [24]. Confidence in the usefulness of peptide array technology for studying signal transduction came from Western blot analysis of lipopolysaccharide-stimulated cells, which corroborated the signals obtained using peptide arrays, as well as from the demonstration that kinase inhibitors affected peptide array phosphorylation patterns consistent with the expected action of these inhibitors [25, 26]. Obviously, employing this kind of peptide array technology for the purpose of investigating cellular properties would allow faster and more inclusive analysis of the changes in cellular metabolism and cellular signaling pathways during successive commitment of these cells during differentiation in comparison with other currently available technologies. 

## 3. Competing Technologies

The predominance of phosphorylation as a regulator of cellular metabolism has enticed many researchers to develop strategies for making descriptions of cellular phosphorylation events. Classically, kinase activity and protein phosphorylation were studied using in-gello kinase assays or Western-blot-based gel shift techniques exploiting the size difference between the phosphorylated and unphosphorylated forms of proteins [27]. These are, however, fairly cumbersome techniques and do not allow the study of large numbers of samples. The situation has improved with the advent of phosphospecific antibodies in late nineties, which recognize the phosphorylated forms of proteins but not their unphosphorylated counterparts. Employing these antibodies phosphorylation events can be detected using classical Western blotting [28] but also in, for instance, Elisa formats to allow high-throughput screening for kinase-activity-modifying compounds [27, 29] or tissue arrays enabling histological analysis of protein phosphorylation in evaluating hundreds to thousands of relevant tissue samples simultaneously [30]. The main drawback remains that only one type of phosphorylation is studied per experiment. Recently, employing multicolour FACS, Irish et al. characterized phosphoprotein responses to environmental cues in acute myeloid leukemia at the single-cell level [31]. The advantage this approach is that the individual variation of cells with respect to the amount of phosphoproteins is assessed and requires very little material, but even the most advanced FACS technology does not allow simultaneous assessment of, typically, more as ten antibodies at one time. This has prompted investigators to explore techniques for studying cellular phosphorylation with little a priori assumptions as to the phosphorylation events involved, with kinome profiling as the main result.

Among the advantages of these approaches is the one that is commercially offered by Kinexus, which uses a multiblot system that relies on sodium dodecyl sulfate (SDS)-polyacrylamide minigel electrophoresis and multilane immunoblotters to permit the specific and quantitative detection of 45 or more protein kinases or other signal transduction proteins at once [32]. When used to its full extent this technology produces almost complete descriptions of cellular phosphoprotein networks, although it still remains fairly labour intensive. Alternatively a proteomic approach may be chosen, which typically consists of a separation of phosphoproteins by, for instance, 2D gel electrophoresis or chromatography, followed by mass spectrometry [33–36]. Steady progress is made in this area and very recently using strong cation exchange (SCX) chromatography at low pH to enrich for tryptic phosphopeptides the first large-scale proteomic profiling of phosphorylation sites from primary animal tissue has been performed [37]. For protein spots that can be detected and unambiguously identified, these approaches provide a powerful way of monitoring the expression and regulation of potentially hundreds of proteins simultaneously, but in practice it is hampered by the fact that the positions of scarcely more than two dozen protein kinases are available on 2D proteomic maps. This reflects the fact, that like most signal transduction proteins, protein kinases are present at very minute levels in cells and are often undetectable by the most sensitive protein stains, whereas procedures based on the purification of phosphopeptides and determination of peptide structure by MALDI are time consuming. The major advantage of this approach is that it is completely unbiased, for instance using microfluidic compact disk technology this approach identified two novel phosphorylation sites in the human mineralocorticoid receptor [38].

A disadvantage of all these approaches is that they are focused on the static determination of the relative concentration of phosphoproteins, but do not address the actual activity of various cellular signalling pathways (a popular comparison is the dashboard of a car where the mileage indicator gives information as to distance travelled but gives no information as to the velocity at which this is occurring; for obtaining the latter information one uses the speedometer) [39].

## 4. Kinome Profiling versus Expression Arrays

**Figure 1:**
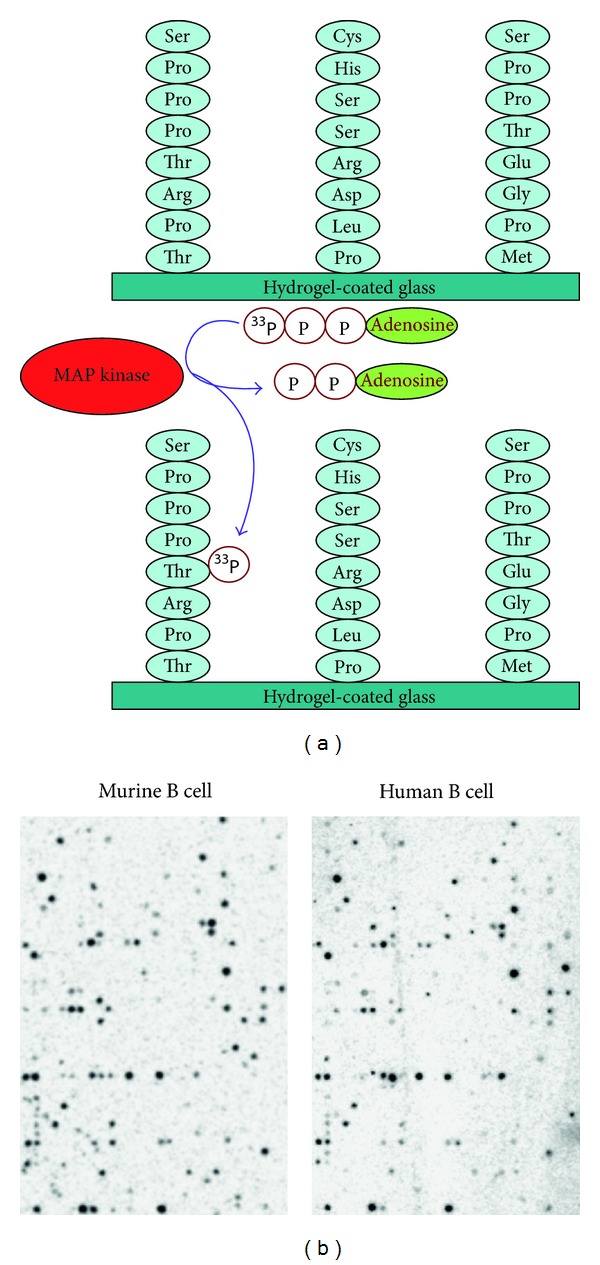
Principle of kinome profiling using peptide arrays. (a) A peptide array consists of peptide consensus motifs for the different kinases in human genome. As an example, the consensus sequences of three different kinases are shown (ISS kinase, MAP kinase, and GS kinase). When incubated with a lysate containing active MAP kinase and radiolabelled ATP, the MAP kinase consensus motif will be phosphorylated with radiolabelled phosphate by MAP kinase without concomitant phosphorylation of the other motifs. Thus, all kinases can be assayed in parallel. (b) We employed this situation to compare phosphorylation patterns by lysates made from B cells from different species and we observed that these patterns were highly similar between species. Furthermore comparison to other cell types showed that phosphorylation of kinase substrates is cell-type restricted and not species restricted and that the divergence in kinase primary sequence between the species is not reflected in differences in substrate phosphorylation.

Despite the complexities in designing peptide arrays [40, 40], an important advantage of metabolic arrays above expression arrays is that the former are nonspecies restricted. Consensus sequences for most kinases are pan-eukaryotic identical (e.g., the nine-amino-acid amino sequence for the cyclin-dependent kinase target Wee1 is identical for yeast, plants, and humans). This offers the advantage that it is possible to directly compare kinase activity in human cells to murine cells and that it is possible to study organisms that have not been completely sequenced. The importance of kinases in regulating eukaryotic protein phosphorylation is widely recognised. Specificity of the kinase reaction as catalysed by individual kinase is dependent on a number of factors, but prominently involves the amino acid context in which the substrate serine, threonine, or tyrosine is placed but other substrates are also recognised by kinase (like, aminoglycoside, choline, and inositol). Despite the large range of substrate classes the overall structure of the kinase domain remains similar. For most typical protein kinases, the exact requirements for this amino acid context for the kinase to recognise its substrate have not been established but apparently involve an 8–12-amino-acid long peptide stretch that fits in a specific groove of the kinase domain [41]. Although kinases show substantial sequence diversity within the eukaryotic kingdom, both substrate sequences and phosphorylation patterns appear remarkably conserved between species (Figure 1). 

## 5. The First-Generation Array

**Figure 2:**
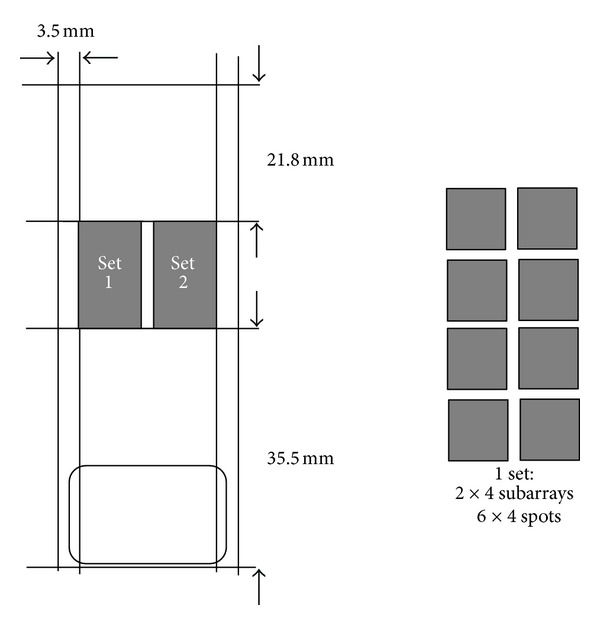
Design of the archetypical first commercial peptide array for kinome profiling from 2003 (produced at that time by Pepscan Systems). The relative small and PKC/PKA biased peptide set precluded wide spread implementation. Its low costs, however, make this chip highly attractive for optimalisation of assay conditions for particular tissue types and for this purpose it is widely used to this day.

As stated, making comprehensive descriptions of cellular signal transduction and metabolism remains exceedingly complicated. This consideration prompted the development of platforms that contain spatially addressed mammalian kinase substrates [42]. The first commercially available platform consisted of 192 peptides providing kinase substrate consensus sequences across the mammalian kinome which were spotted two times, using a biorobotics microgrid spotter equipped with 100-micron split pins. Peptides were spotted onto and subsequently covalently coupled to branched hydrogel polymer-coated glass slide. The final physical dimensions of the array were 19.5 × 19.5 mm, each peptide spot having a diameter of approximately 350 *μ*m, and peptide spots being 750 *μ*m apart (Figure 2).

Confidence in the potential applicability of kinome profiling platforms came from experiments in which a purified kinase was added to this array. If the arrays would measure sensible information, in the presence of ATP this should result in the phosphorylation of the appropriate consensus peptide sequences without concomitant phosphorylation of other peptides (see also Figure 1) and this actually occurred: when arrays were incubated for 90 minutes at 30°C with 25 ng of the constitutively active catalytic subunit of protein kinase A (PKA) and ^33^P-*γ*-strong phosphorylation of limited subset of peptides on the array was detected using phosphor imaging. Analysis of the peptide sequence allowed the extraction of the known most optimal PKA consensus sequence, whereas accompanying phosphorylation of peptides not containing PKA consensus phosphorylation sites was negligible. Incubation of the array with PKA and ^33^P-*α*-ATP did not lead to a detectable signal on the array, demonstrating that spot phosphorylation was a specific binding of the *γ*-phosphate of ATP to the nonapeptides. These results identified the array as useful tool to determine substrate specificity of [24]. This design was also tested in a real biological situation employing actual whole cell lysates and published in the same landmark study describing the previously-mentioned PKA results and included an attempt to make the first comprehensive description of the temporal kinetics of phosphorylation events induced by lipopolysaccharide stimulation [24, 43]. The hope for kinome profiling always included the discovery of novel signal transduction connections and thus this was also attempted for liopolysaccharide signalling. Analysis of the signals obtained on the peptide arrays suggested activation of p21Ras by lipopolysaccharide and this was confirmed by direct measurement of p21Ras GTP levels in lipopolysaccharide-stimulated human peripheral blood mononuclear cells, which represents the first direct demonstration of p21Ras activation by stimulation of a Toll receptor family member [44]. Further confidence in the usefulness of peptide array technology for studying signal transduction came from Western blot analysis of lipopolysaccharide-stimulated cells, which corroborated the signals obtained using peptide arrays, as well as from the demonstration that kinase inhibitors affected peptide array phosphorylation patterns consistent with the expected action of these inhibitors. It was concluded that this first metabolic array is a useful method to determine the enzymatic activities of a large group of kinases, offering high throughput analysis of cellular metabolism and signal transduction [24], although this was overstatement: the PKA and PKC biases substrate set and its overall small number of peptides do not offer true kinome-wide profiling possibilities.

Maybe the main application for this array came (apart from its use for condition optimalisation) from the plant field, which is relatively cash-strapped (making the economy of this particular peptide array slide attractive) and which lacks agents for phosphospecific detection of phosphorylation events on Western blot and an important study was published on the infection of *Arabidopsis* with an avirulent strain of the bacterial pathogen *Pseudomonas syringae* pv. *tomato* in Plant Methods [45], which detected differential kinase activities by measuring phosphorylation of consensus peptides. These days, however, also in this field more advanced formats are used [46].

## 6. The Kinase Array

**Figure 3:**
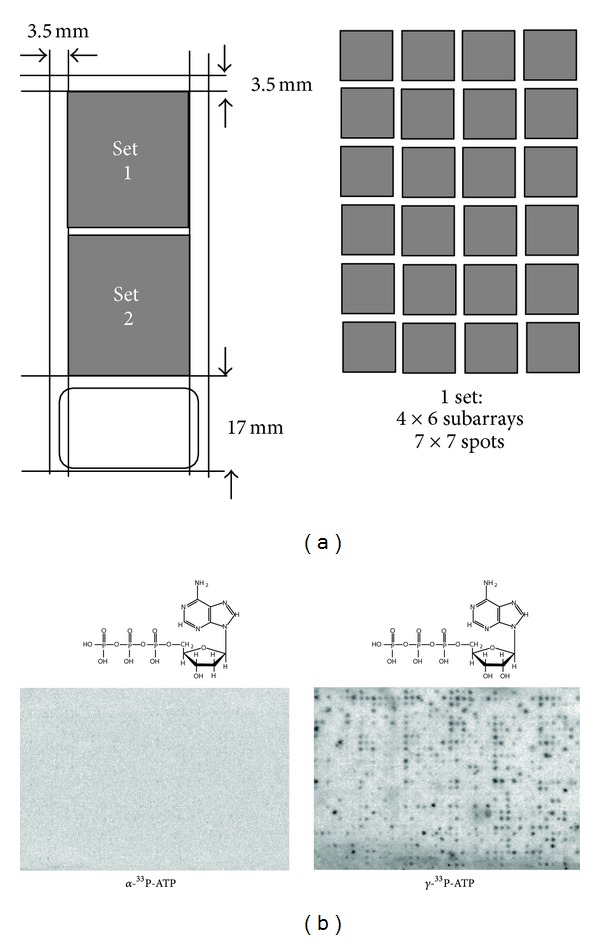
The Pepscan systems produced kinase array. Left.The design includes 1176 substrates that bar a number of spotting controls are derived from the PhosphoBase resource and thus represent peptides that in a bona fide kinase reaction are phosphorylated by a peptide. Right. The reaction involves the covalent transfer of the terminal phosphor group of ATP as arrays efficiently incorporated radioactivity if incubated with cell lysates and ^33^P-*γ*-ATP but failed to do so when incubated with cell lysates and ^33^P-*α*-ATP.

In response to the limitations of the first-generation array, Dr Jos Joore at that time with Pepscan presto decided to increase the number of substrates employing a meritocratic approach, that is, only using peptides proven to act as kinase substrates. To this end the Phosphobase resource ([47–58], http://phospho.elm.eu.org/) was exploited and arrays were produced containing 1176 substrate peptides, which include more-or-less all known human kinase consensus sequences and spotted these in duplicate on glass cover slips to create a second generation of PepChips. These arrays efficiently incorporated radioactivity if incubated with cell lysates and ^33^P-*γ*-ATP but failed to do so when incubated with cell lysates and ^33^P-*α*-ATP, demonstrating that spot phosphorylation was also for these PepChips mediated by a specific binding of the *γ*-phosphate of ATP to the nonapeptides (Figure 3).

Confidence in the usefulness of these arrays for studying cellular signalling was bolstered by a study in which we employed kinome profiling for studying the rapid effects of glucocorticoids (GC) on signal transduction in activated T cells [59–61]. The classical theory of GC action comprises binding to an intracellular glucocorticoid receptor followed by the modulation of transcriptional and translational events. Over the past two decades increasing evidence for rapid, nongenomic effects of GCs on cellular function has accumulated, incompatible with the traditional model. Some effects of GCs occur too rapidly to be explained by genome-dependent processes and these nongenomic effects *in vivo* are clearly shown in various clinical settings. Examples are the rapid response (within 10 minutes) to GCs of an asthmatic patient or the termination of an anaphylactic reaction by GC administration. Hence, human CD4+ lymphocytes were treated with dexamethasone (DEX), a synthetic GC, for 10 minutes and subsequently stimulated for 15 minutes with anti-CD3 and anti-CD28 monoclonal antibodies. Kinome analysis revealed significant alteration of kinomic profiles in activated CD4+ cells upon DEX treatment. Interestingly, DEX treatment resulted in reduced Lck and Fyn enzymatic activity, and this was reconfirmed by *in vitro* kinase assays. Lck and Fyn, members of the Src kinase family, are key players in T-cell receptor signaling. Accordingly, Western blot showed decreased phosphorylation of signaling molecules downstream of the TCR upon DEX treatment, pointing out the rapid effects of GCs on early TCR-signaling events. Thus the PepChip analysis using kinase array, demonstrated rapidly altered kinomic profiles due to DEX treatment and identified Lck and Fyn as novel targets for GC treatment in T lymphocytes. It was concluded that peptide-array-based kinome analysis using this second-generation Pepchip is a powerful novel tool for studying cellular signal transduction [59, 61, 62]. 

The power of this kinome profiling was subsequently shown in various high profile studies, mainly focussing on cancerous disease. Two early studies, published in Cancer Research, used this platform to characterise the changes in the sequence of cellular events leading to adenocarcinoma of the oesophagus [63–66], whereas the other characterised signalling in colorectal cancer [67, 68], but this platform was also used for charting differences in the substrate specificity of related kinases, for example, to characterise the differences between c-Raf and Cot [69] or the phosphorylation target site of DMPK E and lats2 [70] and thus was the first academically truly successful peptide-array-based kinome profiling tool, used in a variety of species (e.g., Guinea pigs [71]). An important recently published study used this platform to characterise the changes in cellular kinome associated with differentiation during haematopoiesis [72], showing that even under unstimulated conditions important differences between cell stages exist, challenging the notion that cellular signalling is mainly reactive. With the advent, however, of more modern better designed platforms its use in recent years has substantially declined.

## 7. Application of the Kinase Array for Nonmammalian Biological Systems

Comparative analysis of genomes has demonstrated substantial differences in the kinome of different eukaryotes. These differences are reflected in highly different number of kinases present in the genome of different eukaryotes (e.g., the *A. thaliana* genome contains 610 apparent kinases, the *H. sapiens* genome exhibits 518 kinases, *D. melanogaster* appears to have 239 kinases, *S. cerevisiae* has 115 kinase genes, and *P. falciparum* exhibits only 65 putative kinases), as well as highly divergent structure. For instance, the plant and unicellular eukaryotes did not show any apparent kinases from the tyrosine kinase group, despite the detection of phosphorylated tyrosine residues in plants, suggesting that tyrosine phosphorylation in these organisms is mediated via other types of kinases as in animals. Strikingly, of the 106 putative kinases in *S. pombe*, based on the primary sequence, only 67 have orthologues in bakers yeast and a paltry 47 have an orthologue in *H. sapiens*. Of the *P. falciparum* kinome, 30% belongs to a group that is called the FIKK family of protein kinases and which is not found in other groups of eukaryotes [73]. Plants contain a group serine/threonine kinases called receptor-like kinases, which is not found in other eukaryotes, but most likely shares a common evolutionary origin with the receptor tyrosine kinases present animals and are thus sometimes collective and called receptor kinases. Whereas fungi such as yeast and *Neurospora* do not appear to have representatives of the receptor kinase group, the slime mold, *Dictyostelium discoideum*, has several examples. Thus the eukaryotic family of protein kinases displays substantial diversity at the genetic level, but much less so at a substrate level. This was exploited by kinase arrays [74, 75], for instance, aiming to define a minimal eukaryotic kinome, using the kinase peptide array. A set of substrates was identified whose phosphorylation by cellular extracts is common to the divergent members of the Animal, Plant, and Fungal kingdoms and may thus be called a minimal eukaryotic kinome (the kinases responsible for phosphorylation of these substrates are implicated in, amongst others, processes like transcription, translation, and cytoskeletal reorganisation) [76, 77]. These results show that the divergence of eukaryotic kinases observed at the level of primary sequence is poorly reflected at level of substrate phosphorylation, which reveals a fairly limited substrate space for the kinase family of enzymes among eukaryotes. Furthermore, the minimal eukaryotic kinome identified suggests the presence of a set of kinase substrates already present in an ancestral eukaryote which has remained essential for eukaryotic life ever since. 

The species-independent nature of this array set up is further highlighted by the success of this platform in plant and even bacterial kinase signal transduction. The regulation of carbon metabolism in plant cells responds sensitively to the levels of carbon metabolites that are available. Exactly which metabolites are sensed is not yet known, but it is evident that high levels of external sucrose and glucose to a certain extent mimics important aspects of carbon sensing in plants including induction of sucrose-1-fructosyltransferase (1-SST), the key enzyme for the synthesis of the storage carbohydrates, fructans. The changes in cellular kinase activity in sucrose-stimulated *Arabidopsis thaliana* employing the peptide arrays containing 1176 different kinase substrates [78] described above and the results suggest a signal transduction cascade involving the sequential sucrose-dependent activation of a receptor-like tyrosine kinase, inactivation of a plant homologue of LKB1and SNRK1 followed by the activation of a MAP kinase-like signalling cassette, in turn activating various effector pathways including a Myt/Wee1 response; the authors concludes that the LKB1/SNRK1/MAP kinase signalling cascade [79] is a major effector in sugar sensing in plants. The same group further pioneered plant kinome profiling [80]. Plants defend themselves against infection by biotic attackers by producing distinct phytohormones and the effects of these on the *Arabidopsis thaliana* kinome were monitored using kinase arrays. Differential phosphorylation of substrates for many kinase families between different phytohormones was observed and evidence for cross-talk was uncovered as well [81]. These days, the PhosphoBase-based array (current commercial supplier is Pepscan Presto) has become the mainstay for kinome profiling efforts in plants [82]. 

Intriguingly, despite the dogma that the kinase reaction is eukaryotic-restricted, also in other forms of life kinases are encountered [83]. Eukaryotic-like serine/threonine protein kinases (STPKs) are present in the *Yersinia* genus (a bacterial group notorious for allegedly causing the bubonic plague), but they have also been identified in the soil microorganism *Myxococcus xanthus* and in human pathogens, such as *Mycobacterium tuberculosis*, which even encodes 11 STPKs. Only two of these (PknG and PknK) are soluble proteins, while the other nine STPKs contain a transmembrane domain. Moreover STPKs have also been identified in *Pseudomonas aeruginosa, Streptococcus pneumoniae, *and in* Staphylococcus aureus*. At least two bacterial equivalents, YpkA of *Yersinia *and PknG of *Mycobacterium tuberculosis*, have been shown to be directly involved in the subversion of host cells during the respective infectious processes.A good characterization of what bacterial kinases may mean has been provided for *Rhodococcus fascians *[84–86]. However, the precise biological functions and substrate specificities of these kinases have not yet been defined and thus are poorly understood. Recently, the kinase array has been applied to the PknB kinase of *S. aureus. *This Gram-positive bacterium is part of the human microbiota, but it can turn into a dangerous pathogen, causing a wide range of infections [87, 88]. The PknB kinase is composed of three extracellular PASTA domains (penicillin-binding domains), a central transmembrane domain, and an intracellular kinase domain. Using peptide microarrays the phosphorylation profile of PknB was determined and showed that PknB is a proline-directed kinase [89, 90]. These results were confirmed by mass spectrometry. The stunning observation that human sequences can be used on a peptide array to determine substrate preferences of a bacterial kinase shows the extreme evolutionary pressure to which kinase substrates are exposed and the elucidation of the nature of this evolutionary selection pressure should reveal important aspects of the nature of life.

## 8. Measuring the Enzymatic Characteristics of the Kinome Reaction: The PamGene Platform

While the first generations of slide-based array technology were being developed, the tediousness of this platform for measuring enzymatic kinetics was more and more felt. The Pamgene company was already employing a platform in which samples are constantly pumped through a matrix and the amount of association to a recipient molecule was assessed fluorescently. This enables kinetic measurements in a 96 wells format and it was realised that using fluorescently labelled antiphosphotyrosine this platform could be used to measure phosphorylation of tyrosine residues on specific peptides. The porous microarray platform from PamGene differs from other microarray formats in that it allows exploitation of features such as speed and the ability for kinetic measurements. The substrate has a 500 times greater surface area than conventional planar arrays, which is especially useful for kinome profile applications adequate measurements of biochemical reactions require nonlimiting access of the enzyme to the substrate. The rapid hybridisation is an asset for kinome profiling as it can distinguish different affinities for substrate binding. This real-time “live” monitoring feature also creates the possibility of studying the effect of changing incubation conditions, for example, temperature, which gives a further separating factor in distinguishing different binding affinities. There currently exists 3 units offered by PamGene based on the same microarray substrate material but different-sized throughput instruments. There are a PamStation 4 (small scale academic research), PamStation 12 (for diagnostics), and a PamStation 96 for compound screening and clinical proteomics. In a landmark study, Sikkema et al. identified global tyrosine kinase activity profiles in 30 pediatric brain tumors: 9 ependymomas, 8 pilocytic astrocytomas, and 13 primitive neuroectodermal tumors (PNETs) of which 10 are medulloblastomas [91–93]. For this study Pamchip tyrosine kinase microarray system consisting of 144 peptides representing key tyrosine phosphorylation sites of proteins known to be involved in signal transduction processes was employed. In all pediatric brain tumors high levels of phosphorylation could be observed on peptides corresponding with phosphorylation consensus sequences for Src family kinases (amongst others). To validate the peptide array data, Src phosphorylation levels were determined by western blotting, confirming Src kinase activation. To determine the role of Src kinases in tumor progression 10 pediatric brain tumor cell lines (6 medulloblastomas, 3 astrocytomas, 1 ependymoma) were subjected to Protein Phosphatase 1 (PP1), a potent Src family kinase inhibitor. Dose-dependent decrease in cell survival was observed in all of these cell lines, indicating a possible therapeutic window for Src inhibitory treatment in pediatric brain tumors. It was concluded that the Pamgene approach represents a new high-throughput approach to generate tyrosine phosphoproteomes, a notion also highlighted by the power of this platform to document changes in kinase activity in knockdown zebrafish by the Den Hertog group, no doubt world leader in this field [94]. Currently its main use lies within the pharmaceutical industries [95], but also various academic studies using the platform have been published [96], documenting, for instance, kinase activity of human brain [97], something which is not yet possible on the Pepchip arrays because of high background due to brain lipids. 

The main drawbacks lie in the relatively poorly developed bioinformatical frame work surrounding the Pamgene approach. Whereas for slide-based peptide arrays various academic groups have developed competing bioinformatical analysis tools [93, 98–100], often highly sophisticated, this is not yet the case with respect to Pamchip results. Furthermore the latter platform has not been reliably validated for serine/threonine applications, bar some specific exceptions [101]. Finally, the affinity of phosphospecific antibodies to specific substrates differs [102–104], which makes it more difficult to interpret results. Advantages lie in its nonradioactive high-throughput format and its robust day-to-day stability. 

## 9. The Kinomics Array

Also in response to increasing competition, the kinomics array was developed by Pepscan Presto. These peptide arrays contain theoretically 1024 (in practice less, because of spotting controls, etc.) different kinase substrates in triplicate. These substrates were selected for their usefulness for understanding signal transduction, which facilitates interpretation as compared to earlier designs. Furthermore, theoretically, phosphospecific antibodies are available for each motif, facilitating interpretation. Disadvantages are the underrepresentation of certain signalling pathways, especially in the TGFb/BMP pathway (crucial, for instance, for understanding colorectal cancer [105–107]), its sensitivity to day-to-day variation (making clustering of large groups of data difficult), and the presence of a fairly large group of hyperspots that always seem to be phosphorylated. Practically, these slides are incubated with cell lysates for 2 hours in a humidified stove at 37°C plus ^33^P-*γ*-ATP or ^33^P-*α*-ATP (control for noncovalent specific binding). Subsequently, the arrays are washed in 2 M NaCl, 1% triton-x-100, PBS, 0.1% tween, and H2O, whereafter arrays are exposed to a phosphoimaging screen for 72 hours and scanned on a phosphoimager (e.g.,a Fuji Storm 860, Stanford, GE, USA). The density of the spots is measured and analyzed with array software, freeware amply being available for the web. For the analysis clustering using the spearman correlation coefficient is calculated for each combination of sets and clustering is performed using Johnston hierarchical clustering schemes. For each peptide the average and standard deviation of phosphorylation is determined and plotted in an amplitude-based hierarchical fashion. If only background phosphorylation is present, this amplitude-based distribution can be described by a single exponent. Thus determining the exponent describing amplitude behaviour of the 500 least phosphorylated peptides should give an adequate description of array background phosphorylation and does so in practice. Peptides of which the average phosphorylation minus 1.96 times the standard deviation is higher as the value expected from describing the background distribution are considered to represent true phosphorylation events.This particular array has three sets of which we have now validated more as 200 purified kinases against peptide arrays, which allows to make highly confident statements as to the kinase activities present in the profiles.

The design has been exploited to answer a variety of biological questions, mainly related to human pathology, for instance, in cancer [108–110], blood coagulation [111–117], and inflammatory disease [100, 118], and multiple other applications are possible as well. A recent study, for instance, employed the platform to delineate the influence of Idh2 mutations on cellular metabolism in glioma, whereas a very spectacular study used the platform to characterize the effects of fear on the human leukocyte kinome employing a Bungy jump approach and the platform is now the workhorse of choice for kinome profiling approaches, as illustrated by the discovery of coilin as a phosphorylation target with neurological implications for the human vaccinia-related [119]. Very recently a successor to this successful platform has been designed and is available through the PEPSCOPE company. Based on the kinomics slide by having been culled from the hyperspots and supplemented with peptides sensing previously underrepresented pathways, this should prove a powerful slide. Nothing, however, has been published yet and thus the field awaits validation of this slide

## 10. Other and Future Platforms

Although not as extensively used as the platform described previously, various other successful platforms are available [120–122], including a well-publishing slide incorporating bovine peptide sequences that has been used successfully in a number of settings, especially in delineating the principles of infectious biology in important cattle species [123–125]. Several studies employ home-made arrays, for example, using the SPOTS synthesis method [126–129] or just the simple phosphocellulose paper binding technique [130, 131] and also the human kinome arrays of JPT Technologies are emerging as a factor in the field [119, 119, 132], but use somewhat older technology. In addition, a system involving PCA oligonucleotide spatially-addressed peptides exist. In this system kinase reactions take place in solution and then are spatially separated on basis of the PCA coding. Protein lysates have also been analyzed using the Luminex immunosandwich [133, 134], a bead-based kinase phosphorylation assay [135], but this is not a kinome profiling platform *sensu stricto*. Nevertheless, this study identified Src as a potential target for invasive cancer, not unlike the results obtained with the Pamgene platform [93]. Finally, solution-based mass-spec-dependent approaches may have substantial value. More specifically, factors limiting value through the current platforms include the use of radioactivity through the use of  ^33^P-*γ*-ATP for detection of phosphorylation events, which poses a regulatory burden and hampers the size of experiments that can be performed in conjunction is expensive compared to stable isotopes; and quickly decays hampering the quantitative comparison between experiments performed on different days; surface chemistry artifacts due to the highly packed peptide spots and inability to do proper enzymatic kinetics; rigid design requirements due to the economic necessity to print arrays in batches; the inability to separate single or multiple phosphorylation events on a single peptide; the absence of the possibility of dual-channel measurement as is now current in, for instance, transcriptome determinations using nucleotides labeled using two different fluorphores to compare two samples; and the requirement to work with subphysiological ATP concentrations. All these factors can be addressed by using solvent-based assays in which mixed-peptide solutions are incubated with cell lysates and ATP (or a stable isotope (e.g., ^2^H-*γ*-ATP) to allow multichannel comparison) and subsequent analysis using Mass Spectrometry. This technology allows identification of the individual peptides based on their unique weight (all amino acids except leucine and isoleucine have different molecular weights) and identification of single and multiple phosphorylation events of these peptides based on the discrete weight changes induced. Introduction of such a technology, however, will require extensive validation as well as assessment of its performance relative to the benchmark technology, *in casu* kinome profiling using arrays and until that time current platforms will remain the option of choice for investigators.
